# Southern Medicine

**Published:** 1860-03

**Authors:** 


### Southern Medicine.

Every paper almost in the South is now filled up with something in advocacy of southern rights, southern dignity and southern duties, all of which seems to be right and proper, until it is made to apply to Southern Medical Institutions, and to the support of medical men educated in the South. Notwithstanding this fact, we have no hesitation in saying, that in no particular has the South suffered more severely than in the deprivation of Southern by Northern schools of floods of money and patronage which naturally and rightfully belonged to her own Institutions, and yet we are to be met with the cry of sectionalism, fire-eating, and the charge of introducing politics into medicine whenever a patriotic impulse in reference to this matter is allowed to take the form of words. We shall not be deterred by any fear of offending what is called the *conservatism* of the country, (more properly speaking, Northern arrogance and Southern *submissionism*,) from speaking out as to what we conceive to be the equal antagonism, which exists between the interests of Northern and Southern medicine, as can be found between any other interests whatever. We have no hesitation in saying that it is as much the imperative duty of Southern people to build up their own medical institutions as to foster and sustain their own manufactories—encourage their literary institutions—support their newspapers, or to do any other act which becomes a patriotic and home-loving people. We think it is time that this kind of politics (if it be such) should be introduced into medicine. It is time that we should cease to be dictated to, by Northern Medical Colleges, or Northern medical men. It is time that we should cease to look to *nationality in medicine*, any more than we should look to a *nationality in politics*. Without any special ill will to the medical profession of the North, and simply imitating their sagacity, we say let there be as few Southern students found in Northern Colleges as Northern students in Southern Colleges. Who shall gainsay this? Independent of our obligations to sustain the struggling efforts which are being made to furnish the demand in this particular among ourselves, we know whereof we affirm in the assertion, that Southern Medical Colleges are furnishing all the facilities which are necessary to, or which will be used to advantage by, the medical student. However much we may approve the course of those who left an unfriendly and inhospitable clime during the past winter, we shall much more honor those who have never set foot upon Northern soil to obtain that which can be just as well furnished at home, so far as the general principles of our science are concerned, and much more successfully and appropriately, as it regards the *peculiarities* of disease found in a southern latitude. What can not be furnished by Charleston, Richmond, Augusta, Savannah, Atlanta, Mobile or Nashville, can surely be found in

New Orleans, where immense hospital facilities, and the greatest possible variety of disease, are spread out without obstruction to the enquiring student, and where some of the first talent of the whole country is congregating in schools which we hope soon to see rivalling even in their classes the great schools of Philadelphia.

